# Linking Synthetic Materials Chemistry to Electrocatalytic Performance

**DOI:** 10.1002/anie.4318027

**Published:** 2026-05-21

**Authors:** Debabrata Bagchi, J. Niklas Hausmann, Tobias Sontheimer, Prashanth W. Menezes

**Affiliations:** ^1^ Department of Materials Chemistry for Catalysis Helmholtz‐Zentrum Berlin für Materialien und Energie GmbH Berlin Germany; ^2^ Strategy Department of Energy and Information Helmholtz‐Zentrum Berlin für Materialien und Energie GmbH Berlin Germany; ^3^ Centre for Future Materials University of Southern Queensland Toowoomba Australia

**Keywords:** AI‐driven materials design, catalyst reconstruction, electrocatalysis, materials synthesis, operando studies

## Abstract

Synthetic materials chemistry is the central foundation for advancing the design of solid‐state electrocatalysts, where control over synthetic properties such as phase, composition, crystallinity, defect density, oxidation state, coordination environment, morphology, particle size, and electrical conductivity determine electrochemical performance descriptors. These descriptors include nature of active sites, number of active sites, mass and charge transport, and the local reaction environment, which collectively govern electrocatalytic peformance (ECP), namely activity, selectivity, and durability. In this review, we highlight the synthetic strategies currently employed in the electrocatalysis literature and show how they enable control over the properties of the in situ‐formed active catalyst and its ECP. After highlighting the state of the art, we discuss how new developments in in situ analytics, data‐driven discovery, and autonomous robotics could further improve the understanding, predictability, reproducibility, and throughput of materials synthesis. With these advancements, synthetic materials chemistry will remain a key driving force for electrocatalyst development.

## Graphical abstract

Synthetic materials chemistry governs electrocatalyst performance through precise control of phase, composition, defects, and morphology, while emerging operando techniques, data‐driven approaches, and automated laboratories are advancing the field toward predictive catalyst design for sustainable energy conversion.

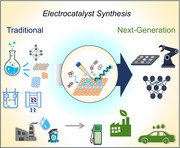

## 1 Introduction

Synthetic chemistry has long been pivotal to the discovery of new materials as it provides the foundation for tuning material properties from atomic to the macroscopic scale [1]. The scope of synthetic processes spans classical solid‐state reactions, nanoscale solution processes, and thin‐film deposition methods, each offering unique opportunities to control electronic, geometric, and surface properties [2, 3]. In the rational design of electrocatalysts, the nature of the (pre)catalyst plays a decisive role, as its structural features determine the type and extent of dynamic reconstruction under electrochemical conditions [4, 5, 6, 7]. These reconstruction processes ultimately define the formation of the catalytically active phase, underlining the necessity of tailoring (pre)catalyst structures through various synthetic techniques. Thus, achieving precise synthetic control is crucial for accessing and stabilizing the true active structure required for efficient electrocatalytic applications [8, 9].

Electrocatalytic performance (ECP) is typically evaluated using a range of descriptors, including activity (overpotential, current density, and turnover frequency, TOF), selectivity toward desired products, Tafel slope, stability under operating conditions, energy efficiency (EE), Faradaic efficiency (FE) for specific products, and durability during extended electrochemical operation [10, 11, 12, 13, 14]. Most of these descriptors are not intrinsic parameters but rather emerge from a complex interplay of catalyst properties at the atomic and mesoscale dimensions. Factors such as crystallographic phase, atomic ordering, oxidation state, electronic conductivity, local coordination geometry, surface termination, defect distribution, porosity, and morphological features directly modulate d‐band center and width, adsorption free energies of key intermediates (e.g., volcano relationships), electrochemically accessible surface area, and the extent of dynamic surface reconstruction [5, 6, 15, 16, 17]. The extent to which these properties define electrochemical performance largely depends on how the (pre)catalyst is synthesized. Synthetic chemistry, therefore, plays a central role in systematically tuning a material's functional landscape. Understanding how catalysts achieve exceptional performance across different electrochemical reactions requires careful examination of their synthetic origins. Mastering diverse synthetic methodologies not only provides researchers with tools to tailor catalyst properties but also enables the design of new materials capable of transforming the future of sustainable energy conversion technologies.

## 2 Synthetic Strategies for Efficient Electrocatalyst Design

**FIGURE 1:**
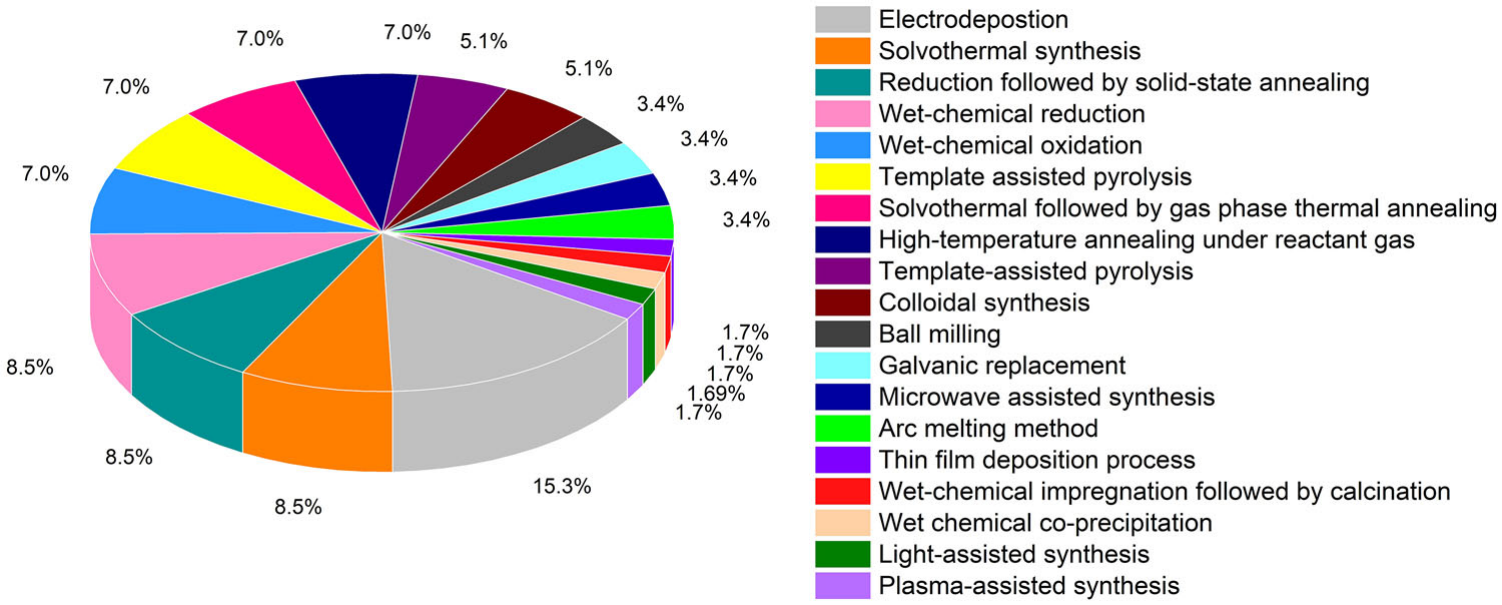
Pie chart showing the number of highly cited publications in electrocatalysis over the past 5 years (2021–2026), categorized by major catalyst synthesis strategies.

To gain insight into the various synthetic methods employed in electrocatalysis, we analyzed highly cited studies from the past 5 years. A pie chart showing the results of this survey highlights the number of studies associated with each major catalyst synthesis methodology, providing a clear idea of the strategies most commonly and effectively used to design efficient electrocatalyst (Figure 1).

**FIGURE 2:**
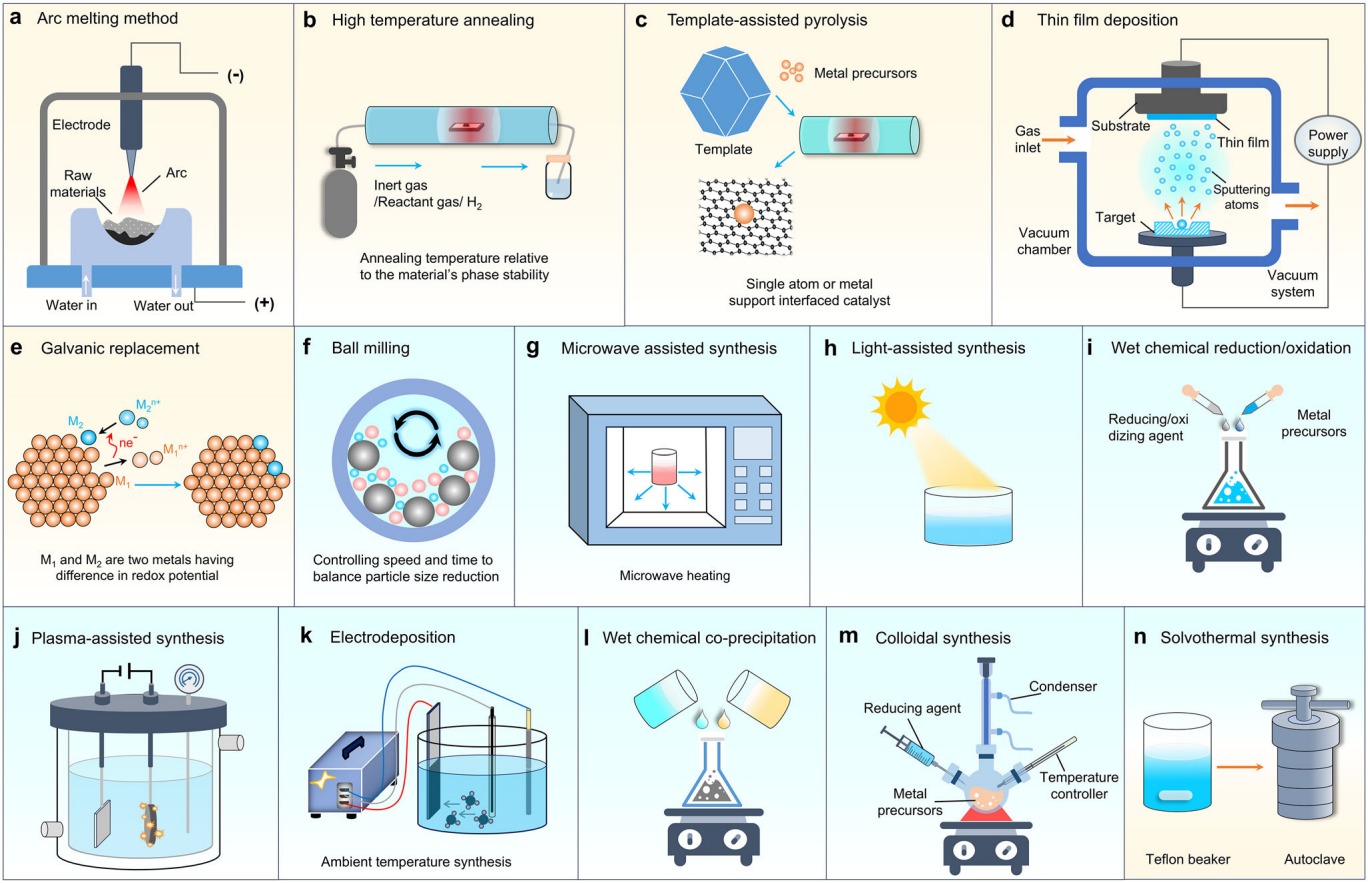
Schematic representation of various synthetic methods employed for the rational design of solid‐state electrocatalysts. The figures display both top‐down and bottom‐up approaches, including (a) arc melting method, (b) high‐temperature annealing, (c) template‐assisted pyrolysis, (d) thin‐film deposition, (e) galvanic replacement, (f) ball milling, (g) microwave‐assisted synthesis, (h) light‐assisted synthesis, (i) wet chemical reduction/oxidation, (j) plasma‐assisted synthesis, (k) electrodeposition, (l) wet chemical co‐precipitation, (m) colloidal synthesis, and (n) solvothermal synthesis. The background colors classify the synthesis strategies according to their dominant reaction control: thermodynamic (beige color), kinetic (cyan color), and hybrid approaches where thermodynamic or kinetic factors could control the formation of the final product.

Figure 2 displays a schematic overview of the various synthetic methodologies employed for the rational design of solid‐state electrocatalysts, illustrating a spectrum from solid‐state high‐temperature (top‐down) to mild, solution‐based approaches (bottom‐up). Table 1 shows a comparative analysis of commonly used electrocatalyst synthesis methods, describing their reaction conditions, advantages, limitations, and the tunability of key catalytic descriptors. The diversity of synthetic strategies for electrocatalyst design arises fundamentally from the nature of the driving forces (e.g., heat, light, chemical potential, electrical potential) applied during synthesis. These driving forces control electron transfer, nucleation, growth, phase stabilization, and crystallization, thereby determining the accessible range of compositions, morphologies, and structural ordering [18, 19].

**TABLE 1:** Comparative overview of synthetic methods for design of electrocatalysts.

Synthesis method	Typical reaction conditions (Thermodynamic/Kinetic control)	Pros	Cons	Types of materials/Examples	Tunability of key descriptors for electrocatalysis	Synthesis‐structure‐performance correlation	Ref.
**Solid‐state synthesis**	Direct mixing of precursors, high *T* (> 700°C), grinding. (Thermodynamically controlled)	Scalable, industrially relevant; simple precursors	Poor control over morphology; large particle size, high energy cost	Perovskites, spinels, mixed oxides, alloys and intermetallics	Crystal phase and electronic structure tunable, but limited control over nanoscale features	Phase purity and controlled bulk stoichiometry provide stable intrinsic TOF, FE, number of active sites; activity modulation primarily arises from electronic structure and chemical environment rather than surface area	[20, 21, 22]
**High‐temperature annealing**	250°C–1000°C, controlled atmosphere (air, Ar, H_2_, N_2_, NH_3_). (Thermodynamically controlled)	Improves crystallinity, conductivity, stability, reduction of oxide phase, nitridation	Loss of surface area; sintering of nanoparticles	Oxides, carbides, perovskites, nitrides, intermetallics, doped carbons	Electronic conductivity and defect chemistry tunable; phase stability enhanced	Phase purity, structural ordering and increases intrinsic TOF and FE while improving long‐term electrochemical stability	[23, 24]
**Template‐assisted pyrolysis**	Organic/inorganic/MOF template + carbonization (600°C–900°C). (Thermodynamically controlled)	Generates porous, hierarchical architectures; heteroatom doping possible	Template removal required; complex processing	MOF‐ or polymer‐derived carbons, N‐doped graphene, porous carbides, Single‐atom catalyst (SAC)	Porosity, surface area, and heteroatom doping tunable; active site accessibility improved	Porosity and surface area enhance mass transport and site accessibility, influencing intrinsic TOF and FE. Graphitization improves conductivity and stability via stronger metal‐support interactions	[25, 26]
**Galvanic replacement**	Aqueous/solution‐phase redox exchange between metals having different reduction potentials. (Thermodynamically controlled)	Generates hollow, porous nanostructures; easy to carry out and can be employed in large scale	Difficult to control stoichiometry; limited to compatible redox couples	Pt‐Ni, Pd‐Cu, Au‐Ni, Rh‐Cu nanostructures	Defect density, strain, and interfacial sites tunable; loading of precious metal, local coordination can be tuned	Redox potential difference between constituents govern phase distribution and lattice strain, tuning the TOF and FE	[27, 28, 29]
**Thin‐film deposition (e.g., sputtering, PVD, ALD, CVD)**	Vacuum deposition; substrate‐specific. (Thermodynamically controlled)	Precise thickness/composition control; excellent uniformity	High cost; equipment‐intensive;	Metal thin films, oxides, nitrides, carbides	Active site exposure, conductivity, and interface engineering highly tunable	Film thickness and growth rate define active site density and transport length, provides TOF and FE‐thickness‐kinetics relationships in model catalyst systems, and enable facet‐dependent studies	[30, 31]
**Ball milling**	Mechanical grinding at room *T*, sometimes with process control agents. (Kinetically controlled)	Solvent‐free, green, scalable; induces defects	Poor control over particle uniformity; amorphization and oxidation risk	Nanostructured alloys, oxides, carbides	Defect density, particle size reduction, and surface reactivity tunable	Milling energy and duration tune defect density and crystallite size, providing non‐linear TOF and FE trends with stability loss at excessive disorder	[20, 32]
**Microwave‐assisted synthesis**	Microwave irradiation, rapid heating, minutes instead of hours. (Kinetically controlled)	Ultra‐fast, energy‐efficient, promotes defect‐rich structures	Limited penetration depth; scale‐up difficult	Metal oxides (NiO, Co_3_O_4_), sulfides, doped carbons, MOF‐based composite	Defect chemistry, surface functionalization, and crystallinity tunable through irradiation time/power	Rapid heating rate promotes defect retention, leading to increased TOF	[33, 34, 35]
**Light‐assisted synthesis**	UV/visible light irradiation, ambient or mild temperatures. (Kinetically controlled)	Provides non‐equilibrium phases, spatial and temporal control, and selective growth	Limited scalability; reproducibility challenges	Metal nanoparticles, oxides, sulfides (e.g., photo‐reduced Pt, Au NPs; defect‐rich TiO_2_)	Control over defect density, particle size, and dispersion of active sites	Photon flux controls nucleation and defect trapping, modulating surface electronic states, FE and TOF	[36]
**Wet chemical reduction/Oxidation**	Solution‐phase reduction (NaBH_4_, H_2_) or oxidation (air, H_2_O_2_). (Kinetically controlled)	Produces nanosized particles; relatively fast	Limited control over size/shape; byproducts often present	Metal (Au, Pt, Ru, Mn, Cu‐based) nanoparticles oxides/hydroxides/borides	Particle size, surface energy, and active site exposure tunable via reductant strength and kinetics	Reducing strength and surfactant regulate nucleation kinetics and surface coordination, producing size‐ and facet‐dependent TOF and FE trends	[37, 38, 39]
**Plasma‐assisted synthesis**	Low‐temperature plasma, ambient or low pressure, short treatment times. (Kinetically controlled)	Rapid synthesis, low thermal budget, high defect generation, surface modification	Equipment complexity, difficult to precisely control uniformity	Defect‐rich carbons, metal foams, oxides, doped graphene, metal/metal oxide, oxygen‐vacancy‐rich oxides	Control over surface defects, heteroatom doping, higher vacancy concentration	Plasma power/exposure tunes vacancy concentration, enhancing TOF and FE; excessive treatment accelerates degradation	[40, 41]
**Electro** **deposition**	Ambient/aqueous electrolyte; applied potential/current density. (Kinetically controlled)	Direct growth on conductive substrates; scalable; controllable morphology and thickness	Limited compositional control; often amorphous or poorly crystalline	Transition‐metal hydroxides (Ni/Fe/Co‐hydroxides), Pt/Pd/Ag/Au‐ or Ni/Cu‐based nanoparticle and few intermetallic	Electronic conductivity via substrate coupling; surface area via deposition parameters; active site density through charge loading	Applied potential, current density, and charge loading directly control nucleation density, enabling quantitative TOF and FE morphology‐selectivity‐stability correlations	[42, 43, 44]
**Wet chemical co‐precipitation**	Aqueous salts + precipitating agent; moderate T (room to 100°C). (Kinetically controlled)	Simple, cost‐effective, scalable	Poor crystallinity; agglomeration unless carefully controlled	LDHs (NiFe, NiMn), oxides, hydroxides, carbonates, phosphates, sulfides, Prussian Blue Analogues	Active site density and porosity tunable via precursor ratio, pH, and precipitation kinetics	Precursor ratio and precipitation rate define cation distribution, governing intrinsic TOF and FE	[45, 46]
**Colloidal synthesis**	Organic solvents, surfactants, reducing agent, controlled temperature (150°C–250°C). (Kinetically controlled)	Excellent size and shape control; monodispersity	Surfactant removal often required; costly precursors	Pt, Pd, Au, Cu, Ni, etc. based nanoparticles, nanorods, nanocubes, alloy, intermetallic	Shape‐dependent surface facets; surface energy and coordination tunable	Growth temperature and ligand chemistry dictate phase, morphology, enabling direct facet‐specific TOF kinetics and FE correlations	[4, 47]
**Solvo/hydro‐thermal synthesis**	Organic/aqueous solvent, high *T* (120°C–240°C), sealed autoclave. (Thermodynamically/Kinetically controlled)	High crystallinity, diverse morphologies	Requires long reaction times; batch‐limited	Metal nanoparticle, Intermetallic, alloy MOF‐derived precursors, oxides, sulfides, etc.	Crystal phase, morphology, surface area, and porosity finely tunable	Reaction temperature and solvent environment regulate phase and morphology, yielding systematic TOF and FE	[8, 48, 49]

Thermodynamically controlled syntheses, such as solid‐state synthesis, arc melting method, high‐temperature annealing, and template‐assisted pyrolysis, are driven by strong thermal or chemical potentials to minimize Gibbs free energy [20, 21, 22, 23, 24]. These processes favor the formation of equilibrium phases such as highly crystalline metal alloys, intermetallics, oxides, nitrides, and carbides with well‐defined stoichiometries and long‐range order. However, the strong driving force usually prevents the stablization of metastable phases and reduces surface area through sintering and grain growth. Among these, template‐assisted pyrolysis and MOF‐derived synthesis mitigate such drawbacks by providing morphological confinement and heteroatom (e.g., B, N, P) incorporation. This leads to hierarchical and defect‐rich carbonaceous matrices suitable for single‐atom catalysts (SACs) (e.g., Ni, Co, Fe, Pt, Ir‐based SACs) or porous composite catalysts [25, 26]. Similarly, galvanic replacement and thin‐film techniques such as chemical vapor deposition (CVD), though chemically rather than thermally driven, also rely on thermodynamic potential differences to achieve equilibrium structures at interfaces and result in compositionally ordered alloys, multimetallic, hollow morphologies, or core‐shell architectures [27, 28, 29]. In contrast, kinetically controlled syntheses, including ball milling, microwave‐assisted crystallization, wet‐chemical reduction/oxidation, electrodeposition, wet‐chemical co‐precipitation, and colloidal synthesis, emphasize reaction‐rate dominance [20, 32, 33, 34, 35, 38, 42]. These routes provide access to metastable, size‐confined, and defect‐rich nanostructures, where the short‐range ordering and non‐equilibrium features play a key role in tuning electrochemical activity. The reaction potential, pH, or strength of the reducing agent defines the nucleation rate and, consequently, controls surface area and active site density. Solvo‐ and hydrothermal methods bridge these two regimes by providing intermediate control via autogenous pressure and solvent polarity.

In addition to conventional thermally driven synthesis routes that often approach thermodynamic equilibrium, externally driven non‐equilibrium strategies such as light‐assisted and plasma‐assisted synthesis have recently gained attention for catalyst design. In these approaches, energy is introduced through photons or ionized gas species, enabling reaction pathways that are not accessible under equilibrium thermal conditions and are therefore predominantly kinetically controlled. Light‐assisted synthesis operates via two primary mechanisms: (i) photochemical pathways, where photon absorption generates excited charge carriers (electrons and holes) that drive redox reactions, and (ii) photothermal effects, where localized heating induced by light absorption accelerates nucleation and growth processes. These mechanisms enable control over nucleation kinetics, defect density, and electronic structure, often under relatively mild bulk conditions (e.g., low average temperature and pressure). For example, plasmon‐mediated excitation in metal nanostructures can induce localized hot‐electron transfer and transient heating, facilitating non‐equilibrium growth and defect formation [50]. Plasma‐assisted synthesis typically employs non‐thermal (cold) plasmas, such as dielectric barrier discharge (DBD), radio‐frequency (RF), or microwave plasmas, where electrons possess much higher energies than the bulk gas. These energetic electrons generate reactive species, including ions, radicals, and excited neutrals, which interact with material surfaces. Due to the limited penetration depth of these species (typically a few nanometers) and their short lifetimes, plasma‐induced modifications are largely surface‐confined. This leads to rapid surface reconstruction, vacancy generation, and heteroatom incorporation without significantly increasing the bulk temperature [36, 40, 41]. Overall, both light‐assisted and plasma‐assisted methods provide routes to kinetically stabilized, non‐equilibrium catalytic structures by decoupling surface reactions from bulk thermodynamic constraints, thereby expanding the accessible design space for functional catalytic materials. This tunability between thermodynamic ordering and kinetic trapping allows the formation of phase‐ and morphology‐controlled nanostructures [48, 49]. Therefore, synthetic approaches can be broadly understood based on thermodynamic or kinetic control, where each type influences key synthetic properties (SPs), which, in turn, determine the fundamental ECP descriptors as discussed in the next section.

In practical catalyst design, however, many high‐performance electrocatalysts are not obtained through a single synthesis process but rather via integration of thermodynamically and kinetically controlled steps. Such hybrid strategies intentionally decouple phase formation from surface and defect engineering, enabling simultaneous optimization of bulk stability and surface reactivity. Representative examples include kinetically driven routes (e.g., colloidal synthesis, wet‐chemical reduction, electrodeposition, or ball milling) followed by controlled thermal annealing, which stabilizes crystal phase and composition while partially retaining non‐equilibrium features such as lattice strain, defect clusters, or size confinement [26, 34]. Furthermore, thermodynamically derived strategies such as solid‐state or template‐assisted pyrolysis are often subjected to post‐synthetic kinetic perturbations (e.g., plasma or light treatment) to introduce surface vacancies, heteroatom redistribution, or metastable coordination environments without altering the bulk phase [40, 51]. Importantly, these combined approaches highlight the necessity of the boundary regimes between thermodynamic and kinetic control, where synthesis outcomes are governed by competing timescales of diffusion, nucleation, and surface reconstruction rather than by a single dominant driving force. For instance, moderate‐temperature annealing, short dwell times, or solvent‐mediated autogenous pressure in solvo/hydrothermal synthesis can preserve kinetically trapped morphologies while allowing partial thermodynamic relaxation of the crystal lattice [52]. Such intermediate regimes are particularly valuable for electrocatalysis, as they enable the stabilization of metastable active motifs under operating conditions while maintaining sufficient structural robustness for long‐term durability. Consequently, the rational integration of thermodynamic ordering and kinetic trapping emerges as a central design principle for scalable and application‐relevant electrocatalytic phenomena.

## 3 Synthetic Control of Electrocatalyst Properties Governs Electrochemical Performance

**FIGURE 3:**
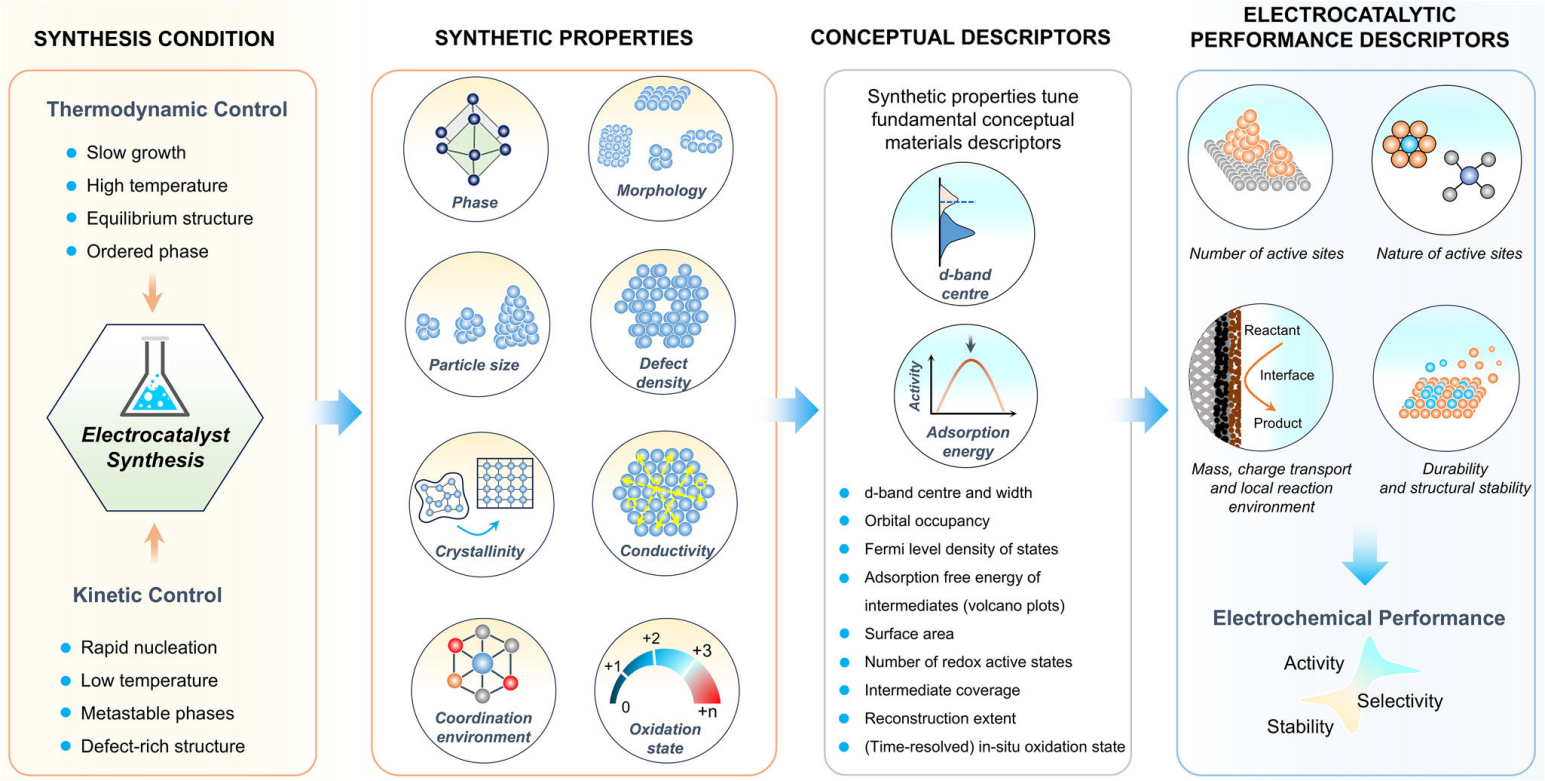
Schematic illustrating the hierarchical link between catalyst synthesis, synthetic properties (SPs), conceptual descriptors, and electrocatalytic performance descriptors (ECPs). Synthetic conditions determine SPs (e.g., phase, morphology, defects, oxidation state, conductivity, etc.), which tune key conceptual descriptors and ultimately govern electrocatalytic performance (active sites, transport, durability, and overall electrocatalytic activity, selectivity, and stability).

The measurable performance of an electrocatalyst arises from a set of ECPs, which directly determine activity, selectivity, and stability. These include (i) nature of active sites, (ii) number of active sites, (iii) mass, charge transport, and the local reaction environment, and (iv) durability and structural stability. Each of these ECPs is governed by distinct SPs such as phase, composition, crystallinity, defect density, oxidation state, coordination environment, morphology, particle size, and electrical conductivity. Importantly, in most systems, the initially synthesized material serves as a (pre)catalyst, whose structure undergoes in situ reconstruction or phase transformation under electrochemical conditions, thereby generating the actual catalytically active phase. Consequently, the SPs indirectly influence not only the initial materials' properties but also the nature and evolution of the active phase during operation. Therefore, understanding and engineering the relationship between SP and ECP is crucial for rational design of efficient catalysts (Figure 3). In heterogeneous electrocatalysis, this relationship has been rationalized using conceptual descriptors that have been successfully linked, qualitatively or quantitatively, to catalytic performance [53]. Such descriptors try to reduce the multiple complex physicochemical material properties to a single most decisive conceptual parameter, such as d‐band center and width, orbital occupancy (electronic filling), Fermi‐level density of states, adsorption free energies of key intermediates (e.g., volcano relationships), electrochemically accessible surface area, density of redox‐active sites, time‐resolved in situ oxidation states, intermediate surface coverage, and the extent of dynamic surface reconstruction [54]. For instance, the adsorption free energies of key intermediates (e.g., Δ*G*_H*_, Δ*G*_CO*_, Δ*G*_N*,_ Δ*G*_OOH*_) often govern catalytic activity through the Sabatier principle, giving rise to volcano‐type relationships across various catalyst materials [55]. Therefore, qualitative activity trends of various catalysts can sometimes be explained with the volcano relation alone, if extrinsic parameters such as surface area remain comparable between the investigated catalysts. The adsorption energy itself will depend in a complex way on multiple synthetic parameters. Consequently, the SP‐ECP relationship can be interpreted as a hierarchical chain: synthetic conditions → SPs → conceptual descriptors → ECP descriptors.

### 3.1 Nature of Active Sites

The intrinsic catalytic activity of a material arises from the local electronic and geometric configuration of its active sites [6]. These atomic‐scale features dictate adsorption energies, intermediate stabilization, and energy barriers for elementary reaction steps. The energetics of these elementary processes collectively determine the TOF of individual sites as well as the overall reaction selectivity. Hence, tuning the coordination and electronic structure of active sites through synthetic control is essential in maximizing the TOF [55]. From a computational perspective, the catalytic behavior of active sites is rationalized using electronic structure descriptors such as the d‐band center or adsorption free energy of reaction intermediates. According to d‐band theory, the position of the d‐band center relative to the Fermi level governs the strength of adsorbate‐metal interactions, thereby controlling the binding energies of key intermediates. Synthetic tuning of alloy composition, lattice strain, or heteroatom coordination can shift the d‐band center and thereby tune reaction energetics [56, 57]. For instance, alloying Pt with transition metals such as Ni or Co downshifts the Pt d‐band center, weakening oxygen adsorption and enhancing oxygen reduction reaction (ORR) kinetics [56]. A major advancement in this direction has been achieved through the development of SACs, where isolated transition‐metal atoms (e.g., Fe, Co, Ni, Mo, Pt, Ir) are stabilized on heteroatom‐doped carbon, oxide, or other conducting matrices [58]. Synthetic routes such as metal organic framework (MOF)‐derived pyrolysis, and atomic layer deposition (ALD) offer exceptional control over the local coordination symmetry (e.g., Fe‐N_4_, Co‐N_4_, Ni‐N_2_C_2_) [58, 59]. Spectroscopic analyses (x‐ray absorption [XAS] and Mössbauer spectroscopy) have revealed that even subtle changes in the local coordination environment induced by precursor chemistry, annealing temperature, or gas atmosphere translate into significant differences in intrinsic activity for reactions such as ORR, hydrogen evolution reaction (HER), and CO_2_ reduction reaction (CO_2_R) [58, 60]. Beyond atomic dispersion, colloidal synthesis, epitaxial deposition, and single‐source precursor decomposition techniques enable control of crystallinity, surface strain, and alloy composition in nanoscale catalysts. In Pt‐Co, Pd‐Cu, Ru‐Te, and Ni‐Ge systems, these synthetic adjustments modify the surface electronic structure and optimize intermediate binding [60, 61]. More recently, the emergence of high‐entropy alloys (HEAs) has provided a chance to modify compositional engineering, where multi‐elemental mixing during synthesis generates a rich diversity of atomic ensembles and active‐site configurations [62, 63]. Hence, the synthetic route directly determines the intrinsic kinetics of active sites, linking atomic‐scale structure with measurable TOF.

### 3.2 Number of Active Sites

While intrinsic activity defines the per‐site efficiency, the number and accessibility of these sites determine the overall current density and EE. This descriptor is governed by structural parameters such as surface area, porosity, dispersion, and connectivity that are again products of synthetic design. In this regard, solvo/hydrothermal methods enable controlled nucleation and growth of active phases directly on conductive supports [48, 49]. A representative example is NiFe‐layered double hydroxide (LDH) grown on Ni foam, a benchmark oxygen evolution reaction (OER) catalyst in alkaline media [64]. The synthesis ensures high dispersion, mechanical robustness, and strong electrical contact, yielding industrial‐level current densities. Similarly, the template‐assisted pyrolysis remains a basis for producing multi‐heteroatom‐doped, hierarchically porous carbon materials. The observed catalytic activity and current density depend not only on intrinsic site activity but also on the electrochemically active surface area (ECSA), which represents the fraction of catalyst surface that actively participates in electrochemical reactions. Synthetic strategies that enhance dispersion, reduce particle size, or generate hierarchical porosity effectively increase ECSA and thereby improve catalytic activity even when intrinsic site activity remains constant [65]. Recent studies on SACs demonstrate that maximizing metal atom utilization can significantly improve the density of catalytically active centers. For example, Fe‐N‐C SAC, with nearly 100% site utilization has achieved ORR activities comparable to Pt‐based catalysts, showing how synthetic control of atomic dispersion directly determines the accessible active‐sites [66]. The use of hard templates (MOF, SiO_2_, MgO) or soft templates (block copolymers, micelles) allows precise control over micro‐, meso‐, and macropore architectures [3, 26]. Such frameworks expose a large number of accessible active sites while facilitating reactant diffusion and product desorption. In metallic systems, controlled annealing of precursor alloys produces highly ordered intermetallic (e.g., Pt_3_Co, PtNi) with defined atomic ensembles and facet orientations [60]. These nanoparticles exhibit enhanced active‐site density and durability compared to random alloys. Thus, the synthetic control of particle size, facet exposure, and porosity dictates the statistical probability of active‐site accessibility, bridging atomic‐scale activity with macroscale catalytic output.

### 3.3 Mass, Charge Transport, and Local Reaction Environment

Efficient electrocatalysis requires not only the maximization of active sites but also the optimization of charge, ion, and mass transport throughout the electrode architecture [60]. Importantly, the local reaction environment is primarily determined by the chemical species and their concentrations near the active site, as well as interactions in the electrochemical double layer, rather than only by charge or mass transport [67]. The synthesis route plays an important role in deciding these transport pathways and influencing the evolution of the local reaction microenvironment during operation. Hierarchical porous carbons, produced via sacrificial templating, sol‐gel processing, or mechanochemical activation, provide continuous networks for electron conduction and reactant diffusion. Such architectures are essential for gas‐phase electroreduction processes such as CO_2_R, where triple‐phase boundaries govern selectivity [10]. Defect‐rich and conductive carbon scaffolds generated by high‐temperature pyrolysis or ball milling ensure both strong metal anchoring and rapid charge transport. In addition, ionomers, ion‐selective membranes, and confined reaction volumes can locally enrich or exclude specific species, thereby tuning selectivity and activity [67]. Advanced thin‐film fabrication techniques, such as electrodeposition, ALD, CVD, and sputtering, further refine these transport properties by achieving ultrathin, conformal coatings of catalytically active materials [30, 31]. These methods minimize interfacial resistance and enable controlled film thickness, critical for high‐current operations in electrolyzer and fuel cells. For example, IrO_2_ and RuO_2_ thin films synthesized by ALD showed superior durability for OER [68]. Moreover, the reaction microenvironment, including local pH gradients, gas solubility, and intermediate concentrations, can be influenced by the synthetic design of the catalyst architecture. Tuning pore distribution and hydrophobic/hydrophilic balance during synthesis can control product selectivity in CO_2_R or suppress bubble accumulation in HER/OER systems [10, 28]. Further strategies include the use of cocatalysts to supply reactants or remove poisons, and surface‐bound ligands or surfactants to modify adsorption and hydrophobicity, thereby directly modulating the local chemical environment [67]. Recent advances increasingly demonstrate that synthetic methodologies can program catalysts to construct highly optimized catalyst‐electrolyte interfaces. An important example arises from interface‐engineered copper catalysts for ECO_2_R, where oxide‐derived Cu structures formed through controlled electrochemical oxidation‐reduction cycles produce grain boundaries, subsurface oxygen species, and nanoconfined pores that significantly tune the local CO coverage and interfacial pH. Such synthetic control over surface reconstruction has been shown to enhance C‐C coupling and increase C_2+_ products [69, 70]. Similarly, defect‐rich catalysts produced through precursor‐mediated pyrolysis can undergo controlled surface reconstruction during electrocatalysis, forming amorphous interfacial phases that stabilize reaction intermediates and enable highly efficient multi‐electron transformations [71]. It has also been reported that synthetic tuning of electronic structure and interfacial coordination can dynamically regulate reaction pathways during electrocatalysis. For example, Ce‐doped CuO_x_ catalysts stabilize dynamic Cu^δ+^ species via Ce^3+^/Ce^4+^ redox buffering, facilitating *CO dimerization during ECO_2_R [72]. Similarly, anion‐substituted NiCo_2_O_4_ spinels enable tunable charge delocalization at Ni active sites, altering urea dissociation pathways and allowing selective N_2_ or NO_x_ formation at current densities up to ∼300 mA cm^−2^ [73]. Interfacial electronic regulation has also been tuned through spin‐state‐modulation, for example, in Pt nanocrystals on Fe‐N‐C supports, where particle‐size‐dependent spin polarization weakens *OH adsorption and delivers high ORR mass activities (∼0.65 A mgPt^−1^) [74]. It has also been found that axial coordination engineering in Co SAC, having dual Co‐C bonds that enhance electron transport and stabilize active sites, promotes the four‐electron ORR pathway and long‐term catalytic durability [75]. Collectively, these examples highlight how synthetic control of electronic coupling, coordination environment, and spin states can govern interfacial charge transport, intermediate stabilization, and catalytic selectivity in electrocatalytic systems. Designing catalysts capable of dynamic interface formation and transport optimization will therefore be critical for achieving electrocatalytic systems operating at industrially relevant current densities.

### 3.4 Durability and Structural Stability

The long‐term durability and structural stability of an electrocatalyst are decisive factors in determining its technological viability, especially under the harsh electrochemical conditions. Durability reflects not only the resistance of a catalyst to physical degradation and chemical dissolution but also its ability to maintain a stable and active phase during extended operation, maintaining initial electrochemical performance. The synthetic chemistry underlying catalyst preparation, composition, crystallinity, interface design, and defect structure plays a central role in dictating how a material evolves under potential cycling and electrochemical stress [4]. It has been established that the catalysts are structurally dynamic systems rather than static solids. Under applied potentials, surfaces and also bulk (in some cases) can undergo electrochemical reconstruction, phase transformation, or amorphization, leading to the emergence of new active states [5, 12]. For instance, transition metal chalcogenides, phosphides, and nitrides (such as Ni_2_P, Ni_3_N, or CoS_x_) frequently convert to corresponding metal (oxy)hydroxide layers during anodic polarization in alkaline OER conditions [6]. Similarly, in CO_2_R or HER environments, restructuring through in situ oxidation, hydride formation, or adsorbate‐induced reorganization alters the surface coordination and electronic structure, modifying both activity and selectivity as operation proceeds [76]. Importantly, a (pre)catalyst that undergoes complete reconstruction can still exhibit exceptional long‐term durability if the in situ formed active phase is thermodynamically stabilized and maintains structural coherence under reaction conditions. Recognizing this dynamic nature, the current focus of materials chemistry is to intentionally control reconstruction rather than prevent it. Synthesis‐guided strategies such as heteroatom doping, phase preconditioning, and core‐shell engineering are increasingly employed to stabilize desirable metastable states while controlling the irreversible degradation. For example, partial substitution of Ni or Co by Fe or Mo can modulate local electronic density, thereby stabilizing the oxyhydroxide phase and suppressing metal dissolution [43]. Similarly, encapsulation of the active phase within protective carbon, silica, MOF, or MgO shells achieved via pyrolysis or templating routes provides both mechanical integrity and chemical passivation without impeding reactant transport [25, 26]. Heterostructure design has also proven effective in improving both activity and durability by fostering strong electronic coupling at interfaces. Combinations such as NiFe‐LDH on Ni foam, or Pt‐Ni intermetallic anchored on conductive carbon supports maintain interfacial coherence and resist detachment under high current densities. Electrodeposition and ALD further enhance structural robustness through precise film thickness control and strong substrate adhesion, essential for long‐term operation in electrolyzers and fuel cells [30, 31, 42, 43, 44]. To unravel these transformations, in situ and operando spectroscopic techniques such as XAS, Raman spectroscopy, and environmental transmission electron microscopy (ETEM) have become very crucial [48, 55]. These tools reveal that durability is an evolving property shaped by the interplay of structure, potential, and electrolyte environment.

## 4 Scalability and Practical Aspects

An important aspect for translating electrocatalysts from laboratory to industrial application lies in the scalability of the synthetic processes, which must satisfy several practical criteria, including low precursor cost, high throughput, minimal solvent consumption, and compatibility with large‐area electrode fabrication [77]. In industrial electrochemical systems, catalysts must typically be produced at the g‐to‐kg scale and integrated onto electrodes with areas exceeding square meters. Recent analyses emphasize that most reported electrocatalysts are synthesized at quantities below 100 mg or electrode areas below 5 cm^2^, highlighting a substantial gap between laboratory demonstrations and industrial requirements for electrochemical technologies operating at current densities exceeding 500 mA cm^−2^. The scalable design of electrocatalysts demands a proper balance between catalyst performance and practical feasibility. Central to this is the abundance and cost of precursor materials, where transition metal, carbon‐based systems, or single‐atom‐based catalysts provide a distinct advantage over noble‐metal catalysts [48]. A similarly critical fact is the scalability of the synthesis. Scalable synthesis approaches such as electrodeposition, hydrothermal/solvothermal growth, thermal annealing, and corrosion‐engineering strategies are increasingly recognized as practical routes capable of bridging this scale gap while maintaining catalytic performance. While colloidal or wet‐chemical methods enable atomic‐level control of active sites, their batch‐to‐batch reproducibility and solvent‐intensive nature limit industrial translation. In contrast, thin‐film‐based or electrodeposition techniques, though less versatile in structural tuning, are inherently more compatible with large‐area, reproducible fabrication [42, 43, 44]. More broadly, the practical scalability of catalyst synthesis methods can be categorized based on their processing energy requirements, complexity, and compatibility with continuous large‐scale manufacturing. Thermodynamically driven techniques such as arc melting and high‐temperature annealing are widely employed for intermetallic and alloy catalysts because they allow bulk production of structurally stable materials with excellent reproducibility. However, these methods generally require higher temperatures and extended reaction times, which can increase energy consumption during large‐scale catalyst preparation. In contrast, kinetically controlled approaches, including electrodeposition, wet‐chemical reduction, and co‐precipitation, offer significant advantages in scalability due to their mild reaction conditions, rapid synthesis kinetics, and direct deposition onto conductive substrates [77].

**FIGURE 4:**
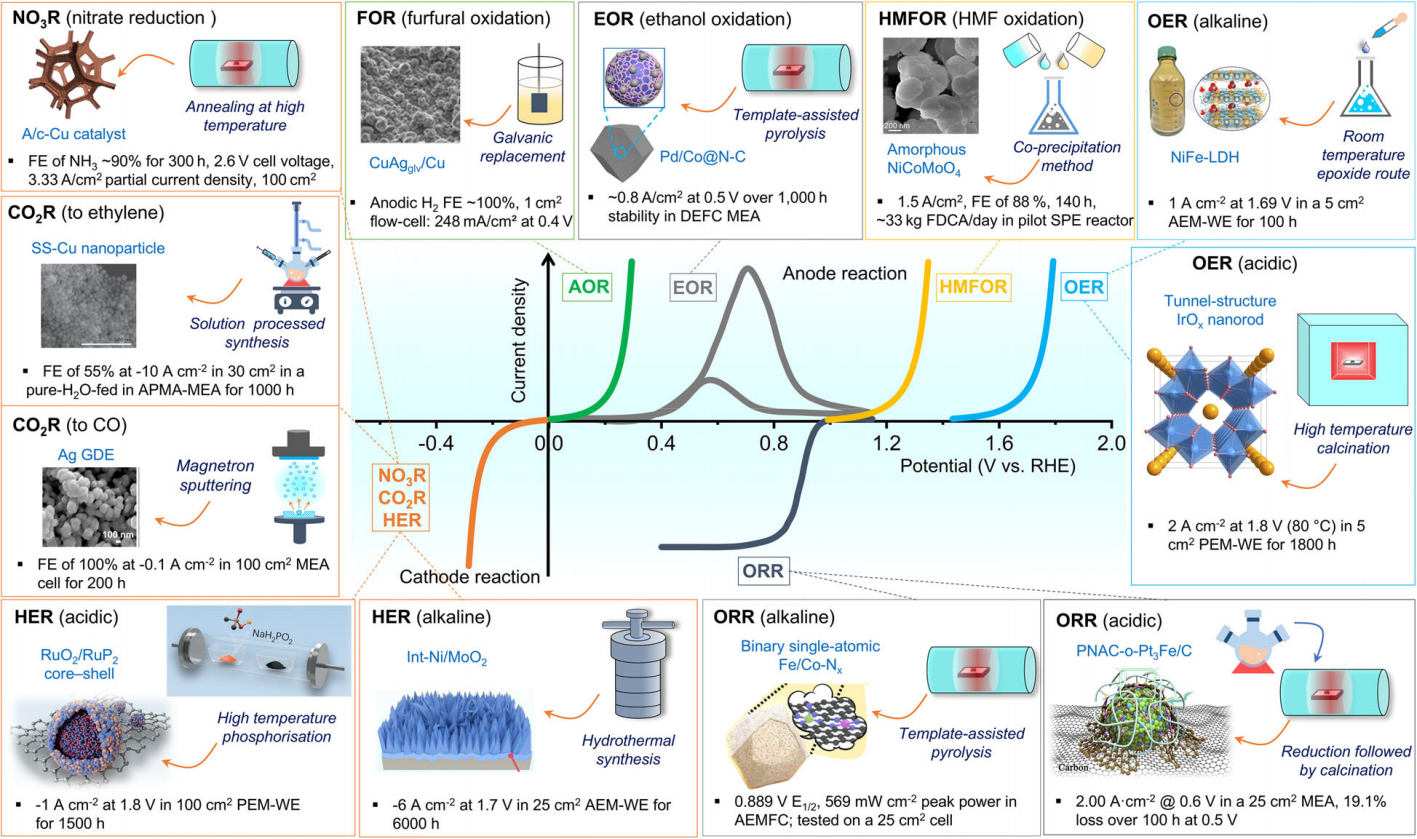
Comprehensive overview of high‐performance electrocatalytic systems for sustainable energy conversion and chemical transformation. The central schematic presents the potential‐dependent polarization curves for a broad spectrum of anodic and cathodic electrochemical processes, highlighting the operational windows for important electrocatalytic reactions. The surrounding panels show how recent advances in synthetic methodologies enable catalysts to operate at practically relevant current densities for various electrochemical reactions. Image for NO_3_RR (nitrate reduction): Copyright 2025, Springer Nature [79]. Image for FOR (furfural oxidation): Copyright 2022, Royal Society of Chemistry [80]. Image for HMFOR (HMF oxidation): Copyright 2025, Springer Nature [81]. Image for EOR (ethanol oxidation): Copyright 2023, Springer Nature [82]. Image for OER (alkaline): Copyright 2024, Springer Nature [83]. Image for CO_2_R (to ethylene): Copyright 2024, Springer Nature [84]. Image for CO_2_R (to CO): Copyright 2025, Springer Nature [85]. Image for OER (acidic): Copyright 2025, Springer Nature [86]. Image for HER (acidic): Copyright 2025, Springer Nature [87]. Image for HER (alkaline): Copyright 2025, Springer Nature [88]. Image for ORR (alkaline): Copyright 2023, Springer Nature [89]. Image for ORR (acidic): Copyright 2025, Springer Nature [90]. All the figures have been reproduced with permission.

However, each class of synthesis strategy presents intrinsic limitations when translated to large‐scale production. For example, arc melting and high‐temperature annealing often promote atom migration and aggregation, which is particularly detrimental for maintaining isolated active sites in systems such as SACs. In contrast, kinetically controlled approaches, including wet‐chemical synthesis, microwave‐assisted routes, and electrodeposition, can stabilize highly dispersed active sites, but frequently suffer from limited batch scalability, poor reproducibility, and challenges in maintaining uniformity at large volumes. Hybrid approaches such as pyrolysis and plasma‐assisted synthesis offer improved control over dispersion and coordination environments; however, they require precise regulation of local parameters (e.g., temperature gradients and precursor distribution), which becomes increasingly difficult under industrial‐scale conditions. Nevertheless, beyond synthesis strategies, different classes of catalysts also face distinct scale‐up challenges. Nanoparticle‐based systems are prone to agglomeration during synthesis and operation, resulting in a loss of active surface area. Alloy and intermetallic catalysts often suffer from phase segregation and difficulties in maintaining compositional uniformity at large scales, particularly under dynamic electrochemical conditions [78]. Oxide and hydroxide catalysts, widely used in alkaline electrolysis, are frequently limited by low electrical conductivity and structural reorganization during operation, which can compromise durability. Furthermore, thin‐film‐based model catalysts, although structurally well‐defined, are inherently constrained by low throughput and high fabrication costs, limiting their industrial scalability. Collectively, these factors highlight that achieving simultaneous optimization of activity, stability, and scalability remains a central bottleneck in practical electrocatalysis [58, 64]. Furthermore, integration with electrode architectures such as membrane electrode assemblies (MEAs) and gas diffusion electrodes (GDEs) necessitates methods capable of producing uniform thin films or hierarchical nanostructures with stable adhesion and reactant/ion/electron transport pathways [10]. The translation of electrocatalyst discoveries from academic laboratories to industrially actionable technologies hinges on the interplay between materials chemistry, scalable synthesis, and reactor‐level integration. As the operating current densities demanded for commercial relevance increasingly exceed the mA/cm^2^ regime, the catalyst architecture, the synthetic route, and the compatibility with large‐area electrodes must be co‐designed rather than optimized in isolation. The representative examples summarized in Figure 4 highlight how recent advances in synthetic methodology have enabled catalysts capable of functioning at practically relevant current densities at various electrochemical reactions, far beyond the limits of traditional screening experiments.

In the field of nitrate reduction (NO_3_R), high‐temperature annealing of copper foil, exemplified by A/c‐Cu systems, indicates the power of simple heat treatment of metal foil in generating structurally resilient catalytically active sites during the reduction process. These electrodes maintain partial current densities of ∼3.3 A cm^−2^ over hundreds of hours for the production of ammonia. Such thermal treatment of metal foils is inherently scalable, as they can be fabricated into large‐area electrodes [79]. For CO_2_R, both colloidal and thin‐film approaches highlight the interplay between activity, selectivity, and manufacturability.

Solution‐processed Cu nanocrystals deliver extremely high ethylene selectivity (FE of 55%) at elevated current densities but may face scale‐up challenges due to batch synthesis, ligand management, and solvent handling [84]. On the other hand, magnetron sputtering produces dense, adherent Ag films directly on gas‐diffusion electrodes, enabling stable CO production (FE of 100%) across tens of square centimeters for hundreds of hours [85]. These comparisons indicate that catalyst design must balance active site tunability with manufacturing practicality, and that scalable deposition methods can also offer a direct pathway to large‐area device integration. Biomass‐derived electrooxidations, such as the oxidation of furfural [80] or 5‐(hydroxymethyl)furfural (HMF) [81], illustrate how room temperature wet‐chemical design or galvanic replacement strategies can meet the dual challenge of activity and resilience for selective product formation. Cu‐Ag catalysts obtained via galvanic simple replacement and amorphous NiCoMoO_4_ phases generated by co‐precipitation form open, defect‐rich networks that resist deactivation while supporting > 1 A cm^−2^ current densities with high FEs over extended operation. These methods indicate aqueous precursors and moderate temperatures, enabling low‐energy, continuous‐flow synthesis and demonstrating how deliberate control over nucleation, multi‐cation incorporation, and porosity directly translates into practical, scalable catalysts for biomass upgrading. In direct ethanol fuel cells and hybrid electrolysis systems, alcohol electrooxidation on Pd/Pt‐based catalysts enables low‐potential oxidation of fuels such as ethanol, methanol, glycerol, and ethylene glycol, providing selective formation of valuable oxidized products alongside efficient energy conversion [91, 92]. For example, template‐assisted pyrolysis yields Pd/Co@N‐C catalyst having hierarchically porous composites with confined metal domains, providing structural stability over thousands of hours in direct ethanol fuel cells [82]. This approach provides how controlled nanoscale architecture fixed within highly conducting graphitic shells prevents coarsening while maintaining electrochemical performance at low precious metal loading.

Scalability is also crucial for the devleoment of OER catalysts. Room‐temperature epoxide‐mediated growth of NiFe‐LDH produces lamellar structures with abundant edge sites, inherently capable of large‐area alkaline AEM electrodes [83]. Because the method employs mild solvents and earth‐abundant precursors, ton‐scale synthesis can be possible. In acidic OER, tunnel‐structured IrO_x_ nanorods, produced by high‐temperature calcination, achieve exceptional durability (> 1800 h at 2 A cm^−2^) in proton exchange membrane water electrolyzer (PEMWE) devices [86]. HER catalysts illustrate the complementary strategies of low‐ and high‐temperature synthesis for acidic and alkaline media, respectively. High‐temperature phosphorization produces RuO_2_/RuP_2_ core‐shell structures with metastable shells intimately bonded to conductive cores, showing continuous operation at −1 A cm^−2^ for over 1500 h in acidic PEM systems [87]. In alkaline HER, hydrothermally synthesized Ni/MoO_2_ nanoneedles grown on Ni foam show that careful crystal engineering at low temperature can tune facet exposure and hydrogen adsorption energetics [88]. Importantly, hydrothermal synthesis is increasingly being used, highlighting how careful design at the atomic and morphological level can yield reproducible, kilogram‐scale materials [78, 93]. ORR catalysis shows the potential of organic‐precursor‐to‐carbonization strategies for scalable electrode design. Single‐atom Fe/Co‐N_x_ catalysts, produced via template‐assisted pyrolysis, achieve high densities of active metal‐nitrogen sites while maintaining a conducting porous carbon framework compatible with binder‐free cathodes in 25 cm^2^ anion‐exchange membrane fuel cells (AEMFCs) [89]. Despite the extremely high atomic utilization and catalytic efficiency of SACs, their practical deployment in industrial electrocatalysis remains limited. In principle, increasing metal loading while maintaining isolated single‐atom dispersion is intrinsically challenging, as higher loadings tend to promote aggregation into clusters or nanoparticles. More critically, the low absolute mass of active sites makes SACs particularly vulnerable to metal leaching under operating conditions, leading to rapid performance degradation. Together with agglomeration and difficulties in stabilizing isolated sites over long‐term operation, these issues pose significant barriers to their integration into industrial electrolyzer systems [94].

Several recent studies have also demonstrated promising scalable strategies. For example, a universal ligand‐mediated approach has been developed for the large‐scale synthesis of transition‐metal‐based (Cr, Mn, Fe, Co, Ni, Cu, Zn, Ru, Pt) SACs, providing the controlled dispersion of isolated metal atoms on various supports while maintaining excellent electrocatalytic ECO_2_R activity [94]. Another notable report demonstrated a general electrodissolution electrodeposition strategy in ionic liquids that directly transforms bulk metals into atomically dispersed catalysts, providing the fabrication of more than twenty SAC systems on centimeter‐scale substrates with excellent HER and OER performance [95]. These advances highlight emerging techniques toward scalable production of SACs. These catalysts deliver > 0.88 V and > 500 mW cm^−2^ in full MEAs. In acidic ORR, ordered intermetallic Pt_3_Fe on porous N‐doped atomically thin carbon (PNAC) formed via reduction and calcination illustrates that elevated thermal treatment can be required when structural resilience is required for sustained high‐current operation in harsh acidic conditions [90]. For practical electrochemical applications, catalyst synthesis must also be compatible with large‐area electrode fabrication and scalable coating processes. Several industrially relevant methods, including sputtering, electrodeposition, and nanoparticle ink deposition, enable the formation of uniform catalyst layers on GDE and MEA. In particular, nanoparticle‐based catalyst inks have attracted considerable attention because they combine the structural tunability of colloidal nanoparticles with compatibility for high‐throughput coating techniques such as spray coating, blade coating, and roll‐to‐roll printing [96]. Such manufacturing approaches are already widely employed in fuel cell and battery industries and provide a viable pathway toward gigawatt‐scale electrochemical technologies [78]. All together, these examples provide a fundamental principle: successful translation of electrocatalysts from lab to device‐level applications. In this context, synthetic materials chemistry serves not only as a tool for performance optimization depending on the various electrochemical requirements but also as a bridge connecting scientific insight with the practical and sustainable chemical manufacturing.

## 5 Advancement in Synthetic Discovery: Beyond Traditional Chemistry

The design and development of efficient, durable, and scalable electrocatalysts have entered a new era, extending beyond conventional synthetic approaches. Historically, materials synthesis has been a cornerstone of innovation in catalyst design, enabling numerous breakthroughs through a combination of empirical knowledge, experimental insight, and iterative optimization. These approaches have provided a deep understanding of structure–activity relationships, phase evolution, and active site engineering, laying the foundation for modern electrocatalysis. Looking forward, the integration of predictive modeling, advanced characterization, and intelligent synthesis strategies promises to enhance the precision, efficiency, and scalability of catalyst development, enabling the rational design of next‐generation materials that meet the growing demands of energy and environmental applications.

### 5.1 In Situ/Operando‐Guided Synthesis

In situ and operando techniques have transformed our understanding of dynamic changes in catalysts during reactions. Now, their application during catalyst synthesis could provide valuable real‐time insights into active site formation and structural evolution. Traditional synthetic control often fails to capture dynamic transformations: (pre)catalyst restructure, evolution of surface terminations, leaching of active metals, and phase transformations induced by electrochemical bias and harsh pH conditions [4, 6, 9]. In situ/operando‑guided synthesis provides dynamic pathways to program catalyst structure and active sites beyond static design approaches. For example, operando XAS, coupled with in situ attenuated total reflectance surface‐enhanced infrared absorption (ATR‑SEIRAS), has revealed a potential‑driven two‑step restructuring of carbon‑supported Fe SAC into their actual active sites during electrochemical N_2_ to NH_3_ conversion, providing direct insight into active site formation under bias that ex situ synthesis alone cannot capture [97]. In another case, operando XAS was used to monitor and program activation pathways of Ni_x_Fe_1‐x_S_2_ precatalysts for industrial OER in alkaline media, controlling surface reconstruction and resulting in improved durability at 1 A cm^−2^ by guiding the synthetic activation process itself [98]. Fast operando XAS tracking of silver nanocrystal evolution during ECO_2_R has shown how in situ defect formation correlates with near‑100% FE and high current density, suggesting defect‑centric synthetic strategies guided by operando findings [99]. Moreover, advanced operando liquid‑cell transmission electron microscopy (TEM) combined with spectroscopy has directly visualized the dynamic morphological transformation of CuO precatalysts into active Cu structures rich in grain boundaries and active sites for C_2+_ products, illustrating how synthesis intermediates and transformation pathways can be guided toward desired architectures [100]. Techniques such as Raman, probe‐based technique (scanning electrochemical microscopy [SECM]), scanning ion conductance microscopy (SICM), electrochemical atomic force microscopy (EC‐AFM) or TEM, when coupled directly into the synthesis process, provide real‐time information on local coordination, oxidation state, phase transformation, grain boundaries, strain or defect generation. For example, during microwave‐assisted synthesis of Ni‐Fe or Ni‐Co hydroxides, real‐time monitoring of phase crystallization can direct heating cycles to maximize defect density required for OER. Similarly, in template‐assisted pyrolysis of MOF, operando monitoring can ensure controlled carbonization and retention of heteroatom‐doped active sites, thereby tuning selectivity for CO_2_R, ORR, or organic oxidation reaction (OOR) to desired products. This capability transforms synthesis from static, endpoint‐oriented methods into dynamic, adaptive routes for material discovery [12, 48, 76].

### 5.2 From Traditional Chemistry to Autonomous Laboratories

**FIGURE 5:**
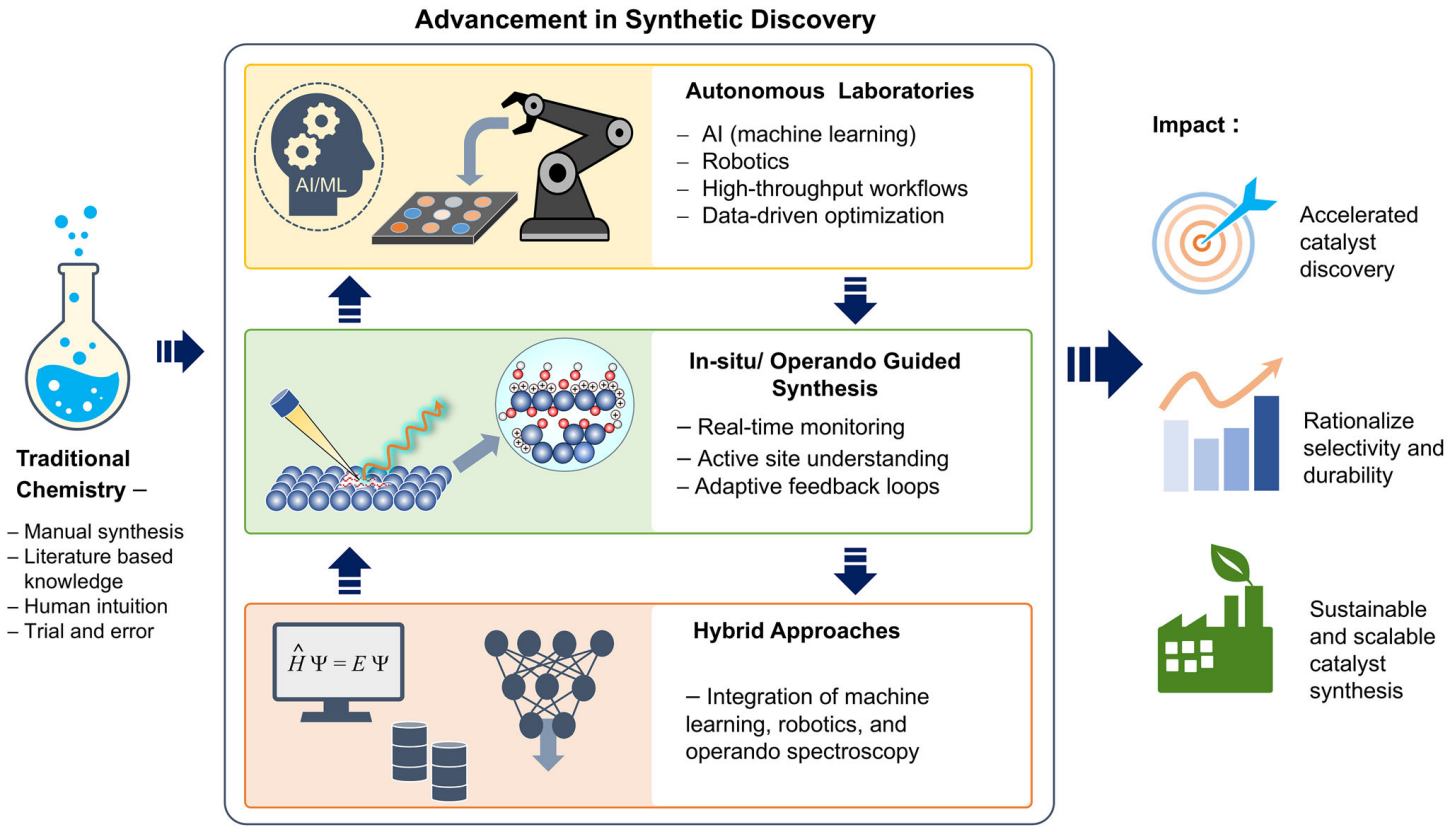
Conceptual illustration of next‐generation electrocatalyst synthesis, depicting the evolution from traditional human intuition‐ and logic‐driven chemistry to autonomous, in situ/operando‐guided, and hybrid approaches. Integration of AI, robotics, real‐time feedback, and sustainable design enables accelerated discovery, tunable selectivity, and advanced applications in electrocatalysis.

Conventional synthesis methods are powerful in tailoring morphology, porosity, crystallinity, or conductivity. However, despite their utility, these approaches remain limited by human‐driven parameter selection and lack the ability to rapidly optimize large compositional and structural varieties. The advancement in autonomous laboratories, integration of robotics, high‐throughput screening systems, and artificial intelligence is extremely important for future catalyst design (Figure 5] [101, 102]. Notably, an autonomous laboratory platform integrating robotics, machine learning, and computational data recently achieved the successful synthesis of 41 previously unreported inorganic compounds within just 17 days, providing the excellent potential of AI‐guided synthesis approaches for facilitating materials discovery [1]. In this case, the synthesis is no longer limited by a large number of trial experiments; instead, machine learning algorithms guide robotic systems to explore and tune reaction conditions. For instance, instead of laboriously screening hundreds of wet chemical reactions, an autonomous platform can execute and produce thousands of electrodepositions or thin film depositions rapidly on optimal compositions with desired activity and durability. A recently developed multimodal robotic discovery platform integrating large‐scale machine learning models, automated catalyst synthesis, and high‐throughput electrochemical testing platform explored more than 900 catalyst compositions and performed over 3500 electrochemical experiments within 3 months, providing the identification of a complex octonary Pd‐Pt‐Cu‐Au‐Ir‐Ce‐Nb‐Cr multimetallic catalyst for the formate oxidation reaction. The optimized catalyst exhibited ∼9.3‐fold improvement in cost‐normalized activity compared with conventional Pd‐based catalysts, highlighting the capability of autonomous systems to navigate high‐dimensional compositional spaces that are impractical to explore using conventional synthetic approaches [103]. A high‐throughput workflow integrating combinatorial synthesis with machine‐learning optimization, provides rapid identification of optimal multi‐element transition‐metal catalysts for OER from a vast compositional space [104]. More broadly, recent perspectives on autonomous catalysis research highlight that human‐AI‐robot collaborative platforms can integrate automated synthesis, real‐time data acquisition, and machine‐learning‐guided decision making to enable closed‐loop optimization of catalytic systems. However, despite these promising developments, several challenges remain, including the integration of operando characterization results into autonomous synthesis loops, the development of structure‐performance descriptors for guiding machine‐learning models, and the translation of high‐throughput discoveries into scalable catalyst manufacturing and device‐level electrochemical validation. Importantly, the current capabilities of robotic platforms are mainly limited to the automation of relatively simple and well‐defined operations, such as solution mixing, precursor dosing, or thin‐film deposition. As a result, these systems are highly effective simplified synthetic frameworks, but they remain limited in executing complex, multistep synthesis and performance testing protocols that often require human intervention and expertise [105]. Consequently, while human‐driven experimentation enables the design and execution of more sophisticated synthetic strategies at lower throughput, robotic approaches prioritize throughput over synthetic complexity. Future advances in robotic control, modular reactor design, and adaptive feedback algorithms are expected to progressively expand the scope of automatable chemistries, providing the integration of complex synthesis pathways into autonomous workflows. Collectively, these emerging studies illustrate how robotic experimentation, high‐throughput synthesis, and machine‐learning‐driven optimization can expand the accessible catalyst design space and accelerate electrocatalyst discovery beyond conventional synthetic routes.

### 5.3 Hybrid Approaches: Integrating Computation, Machine Learning, and Synthesis

While autonomous laboratories and operando understanding provide excellent experimental control, the integration of computation and machine learning makes catalyst design more predictive. Density functional theory (DFT) has long guided the understanding of d‐band tuning and adsorption energies. Machine learning algorithms, trained on existing DFT databases and experimental results, can rapidly predict descriptors such as conductivity, binding energies, or stability windows across vast material types [1, 55]. Hybrid approaches that integrate computational prediction with robotic synthesis and operando validation will become increasingly important. As an example, a computational model may identify that doping NiOOH with Fe optimizes oxygen binding energy for OER in alkaline conditions. A robotic platform can then synthesize hundreds of Fe‐Ni hydroxide combinations using wet chemical co‐precipitation, thin film catalyst or electrodeposition, while operando x‐ray absorption validates the stabilization of Ni^(3‐4+)^/Fe^3+^ sites under bias [22, 43]. This kind of workflow improves the discovery of catalysts not only for OER, ORR, and HER but also for complex reactions such as CO_2_R, OOR, where tuning adsorption energies of multiple intermediates (COOH*, CHO*, OCCO*) determines selectivity.

## 6 Conclusions

Synthetic materials chemistry plays a crucial role in determining the activity, selectivity, and stability of electrocatalysts. The comparative evaluation of various synthesis methodologies reveals that thermodynamically driven routes (e.g., solid‐state, high‐temperature annealing) produce highly ordered and stable phases, whereas kinetically controlled approaches (e.g., wet‐chemical, electrodeposition) enable the formation of metastable, defect‐rich, and highly active nanostructures. Balancing these regimes through hybrid or template‐assisted strategies allows precise tuning of coordination, morphology, and electronic features, thereby linking SPs directly to catalytic descriptors and performance. Moreover, synthetic design governs in situ structural evolution and reconstruction, transforming dynamic behavior into an advantage for achieving long‐term stability. As the field advances, the integration of in situ/operando‐guided synthesis, autonomous laboratories, and data‐driven modeling will enable predictive and adaptive material design. However, fully integrated autonomous platforms that combine synthesis, operando characterization, and high‐throughput screening remain difficult to achieve, especially for real‐time feedback and closed‐loop optimizations. The synergy of these emerging approaches with sustainable chemistry principles will accelerate the discovery of durable and efficient catalysts for future energy conversion and the decarbonization of the chemical industry.

## Author Contributions

**Debabrata Bagchi**: investigation, validation, writing – original draft, formal analysis. **J. Niklas Hausmann**: writing – review and editing, validation, investigation. **Tobias Sontheimer**: investigation, methodology, writing – review and editing. **Prashanth W. Menezes**: conceptualization, validation, visualization, supervision, writing – review and editing, resources, project administration, funding acquisition.

## Conflicts of Interest

The authors declare no conflicts of interest.

## Data Availability

Data sharing is not applicable to this article as no datasets were generated or analyzed during the current study.
